# ‘*Science is only half of it*’: Expert perspectives on operationalising infectious disease control cooperation in the ASEAN region

**DOI:** 10.1371/journal.pgph.0000424

**Published:** 2022-05-04

**Authors:** Anna Durrance-Bagale, Manar Marzouk, Aparna Ananthakrishnan, Michiko Nagashima-Hayashi, Sze Tung Lam, Manit Sittimart, Natasha Howard

**Affiliations:** 1 Saw Swee Hock School of Public Health, National University of Singapore and National University Health System, Singapore, Singapore; 2 London School of Hygiene and Tropical Medicine, London, United Kingdom; 3 Health Intervention and Technology Assessment Program, Ministry of Public Health, Nonthaburi, Thailand; Management Sciences for Health, UGANDA

## Abstract

Governmental awareness of the potential spread of infectious disease, exemplified by the current Covid-19 pandemic, ideally results in collective action, as countries coordinate a response that benefits all, contributing expertise, resources, knowledge and experience to achieve a common public good. However, operationalising regional cooperation is difficult, with barriers including lack of political will, regional heterogeneity, and existing geopolitical issues. We interviewed 23 people with regional expertise focusing on Asia, Africa, the Americas and Europe. All interviewees held senior positions in regional bodies or networks or had significant experience working with them. Operationalisation of a regional infectious disease body is complex but areas interviewees highlighted–organisational factors (e.g. integration and harmonisation; cross-border issues; funding, financing and sustainability; capacity-building; data sharing); governance and diplomacy (e.g. building collaborations and partnerships; communication; role of communities; diplomacy; leadership; ownership; sovereignty; political commitment); and stakeholders and multilateral agreements–will help promote successful operationalisation. The international infectious disease community has learned valuable lessons from the Covid-19 pandemic, not least the necessity of pooling human, financial and technological resources, constructing positive working relationships with neighbours, and sharing data. Without this kind of regional cooperation, infectious diseases will continue to threaten our future, and the next pandemic may have even more far-reaching effects.

## Introduction

The potential spread of infectious disease is one of the most serious threats to global wellbeing, as demonstrated by the current Covid-19 pandemic that has led to the loss of lives, livelihoods and freedoms in most countries [1, 2]. Ideally, national governments would work together through enlightened self-interest, recognising that their approach to global pandemics affects not just themselves but also neighbouring and more distant countries. This awareness should result in network building and collective action, as countries coordinate responses that benefit all, contributing expertise, resources, knowledge and experience to achieving a common public good [2–4]. However, such multilateral, regional cooperation is difficult to implement, with barriers including lack of political support, as governments focus on domestic issues rather than considering global commitments that are less likely to be vote winners; regional heterogeneity–countries may be contiguous but have very different economic, cultural and political outlooks; and geopolitical issues that may complicate attempts by countries to cooperate with regional neighbours [5].

Governments may not be willing to share data, or even formally admit to an issue in their country. This was seen with Covid-19 in Tanzania, when the government denied the country had any cases and refused to distribute vaccines that were not researched and manufactured in Tanzania [6–8]. This denial not only affected health-workers in the country, who had little access to personal protective equipment for a pandemic that was unacknowledged, but may also have damaged an effective regional response to Covid-19 as Tanzanian citizens remained unvaccinated [6]. Alternatively, South Africa’s transparent and rapid response to detection of the Omicron variant led to immediate travel bans by UK, US, Israeli and other governments, resulting in travel cancellations and significant revenue loss for South Africa [9]. Shortly afterwards, the Omicron variant was detected in many other countries, demonstrating the ineffectiveness of travel bans to curb infectious disease spread [10].

Regional organisations and networks, such as African and European Centres for Disease Control (CDC), and multilateral organisations such as the World Health Organization (WHO), are essential to coordinating responses to potential infectious disease threats and facilitating regional cooperation, providing guidance and funding to control any disease spread [2]. Such coordinated responses would reduce economic and staffing burdens on individual countries, as countries share resources, while promoting a coherent and unified plan to address disease spread within and across countries. Collaboration may also help ensure that resource-poor countries are not unduly disadvantaged, as, for example, lack of equitable access to vaccines has been an issue in the current pandemic [11–13]. Without a binding agreement on such access, driven by governments prioritising their own citizens, and pharmaceutical companies choosing to preferentially supply wealthier countries, both health status and economies in poorer countries continue to be disproportionately threatened by the Covid-19 pandemic [14].

Despite being the focus of several infectious disease outbreaks, including severe acute respiratory syndrome and highly pathogenic avian influenza in 2003 [15, 16], the member states of the Association of Southeast Asian Nations (ASEAN) did not have a regional infectious disease coordination and response body until the 12 November 2020 announcement on establishing the ASEAN Centre for Public Health Emergencies and Emerging Diseases (ACPHEED) [17]. There is much work to be done. Planning for future epidemics or pandemics, and addressing current ones, will be difficult, compounded by different governance structures, languages and income levels [18, 19]. We previously explored the literature on operationalisation of a regional infectious disease control body to inform development of a regional body in ASEAN [20], finding key drivers for success included ensuring that context-specific factors were sufficiently considered, that major stakeholders were included in planning from inception to promote buy-in, and that capacity building was ongoing to ensure sustainability [20].

This study goes further, aiming to examine perspectives of experts working with regional disease control bodies on how to operationalise regional infectious disease control cooperation and inform ACPHEED operationalisation.

## Materials and methods

### Ethics statement

The Saw Swee Hock School of Public Health Departmental Ethics Review Committee in Singapore (reference SSHSPH-145) and the Institute for the Development of Human Research Protections institutional review board in Thailand (reference 040–2564) provided ethics approval. Interviewees were asked to sign a consent form or give verbal consent confirming that they had read the study information sheet and agreed to participate before interviewing began.

### Study design and research question

We conducted a qualitative study, using semi-structured interviews with experts on regional disease control. Our research question was: ‘*What lessons can experts provide for operationalising an infectious disease control body in the ASEAN region*?’

### Sampling and recruitment

We used a combination of purposive and snowball sampling. First, we developed a ‘seed list’ of 29 experts covering a range of geographical areas and roles, including representatives of organisations and networks, as identified in a recent scoping review [20], and advocates of regional health collaborations identified from research and policy literature. All were contacted by email, with 13 not responding and five refusing to be interviewed due to time constraints or perceived lack of expertise. Second, we asked each interviewee to suggest additional potential interviewees. We continued this process until no new information was being shared and we judged we had reached saturation. All interviewees were English-speaking infectious disease experts working in one or more ASEAN countries or for a regional disease control body.

### Data collection

The topic guide covered interviewees’ current role and work supporting infectious disease control, experiences of previous national or regional health emergencies and how these shaped the guiding principles for their regional health body, roles of multiple stakeholders, and barriers/enablers to establishing the body (e.g. political commitment, financing).

Interviews lasted 30–60 minutes and were conducted remotely in English using Zoom software (Zoom Video Communications Inc, San Jose) and audio-recorded with automatic transcription enabled. Each interviewer then reviewed and corrected their transcripts. All interviewers had post-graduate training in interpretivist social science research.

### Analysis

ADB and MM analysed interview transcripts with NVivo 12 data analysis software (QSR International Pty Ltd. Version 12, 2018) using a six-step reflexive thematic analysis, as delineated by Braun and Clarke [21] and Byrne [22]: (i) data familiarisation; (ii) generating initial codes; (iii) generating themes; (iv) reviewing potential themes; (v) defining/naming themes; and (vi) synthesising findings. All authors additionally discussed and agreed themes iteratively.

## Results

### Interviewee characteristics and themes

Table 1 shows characteristics of our 23 interviewees, four women and 19 men. Interviewees classed their regional expertise as primarily focusing on Asia (n = 14), Africa (n = 5), the Americas (n = 3), and Europe (n = 1). All interviewees held senior positions in regional bodies/networks or had significant experience working with one.

**Table 1:** Interviewee characteristics.

Code/Region	Gender
Africa1	Male
Africa2	Male
Africa3	Female
Africa4	Male
Africa5	Male
Americas1	Male
Americas2	Male
Americas3	Female
Asia1	Male
Asia2	Male
Asia3	Male
Asia4	Male
Asia5	Male
Asia6	Female
Asia7	Male
Asia8	Male
Asia9	Male
Asia10	Male
Asia11	Male
Asia12	Male
Asia13	Male
Asia14	Male
Europe1	Female

We organised findings under three overarching themes: (i) governance and politics; (ii) organisation and management; and (iii) external partnerships and engagement (Table 2).

**Table 2:** Major themes and sub-themes.

Themes	Sub-themes
Governance and politics	Leadership and diplomacy
	Ownership and sovereignty
	Partnership development and collaboration
	Political commitment and data sharing
Organisation and management	Integration and cross-border challenges
	Financing and sustainability
	Internal communication and capacity-building
External partnerships and engagement	Multilateral partners
	Public engagement and external communications

### Governance and politics

Sub-themes included: (i) leadership and diplomacy; (ii) ownership and sovereignty; (iii) partnership development and collaboration; and (iv) political commitment and data sharing.

#### Leadership and diplomacy

Many interviewees discussed how organisation of a regional body could take advantage of each country’s strongest attributes, to promote information sharing and capacity building among members, and ensure a governing body included representatives of each member country.

*‘I think it can effectively work*, *if it is governed by a group of the people from each country—other than in one individual and he’s the known decision-maker*. *That thing becomes difficult somehow to implement*. *So if you think about that part like maybe team*. *And when the responsibility is shared*, *along with the authority*, *then people become more responsive*. *And if you just allow one person to be authoritative*, *and the others are just to follow it*, *then I think my*, *my thinking is that*, *that it somehow reduces the*, *the potential of the recipient to respond*.*’* [Asia12]

One way of encouraging participation is to leverage each country’s specific strengths and share leadership.

*‘We have a rotation of the chairmanship*. *So*, *the countries are taking the equal chance to lead the network*. *And also*, *we have identified the strategy*. *So*, *each country have to lead each strategy*, *so they don’t need to compete each other […]*. *So*, *that might be the equal ownership*.*’* [Asia1]

*‘We have monthly executive board meetings […] with nine members all together with a rotating chairmanship*. *Each year*, *it goes to one country*, *and all the decisions are done in consensus between the three countries*, *and they choose their priorities*, *what they want to work on*, *what’s important for their countries*.*’* [Asia8]

One interviewee recommended strong recognised leadership, to consolidate collaboration and promote cohesion.

*‘It really is starting with setting up the spirit of the collaboration*, *people need to leave their uniforms at the door*, *leave your egos at the door and join the collaborative*, *make that work*, *and that becomes…you know*, *the spirit of it*, *it often is*, *a few individuals can really make a difference*, *if you have a strong leader that has the charm and diplomacy that can actually pull things together*, *that can really have a big difference*.*’* [Asia4]

Interviewees described the Asia region as particularly heterogeneous, with one suggesting this might cause issues if someone took charge of decision-making.

*‘Africa’s […] heterogeneous of course*, *but there’s that continental identity*, *Asia doesn’t really have that*. *China which is its own superpower*, *India which is its own power and all those conflicts between those players that would resist someone being in charge*.*’* [Asia4]

The key importance of diplomacy was put succinctly by one interviewee:

*‘Lesson number one is*, *science is only half of it*. *The other half is non-scientific issues*, *right*, *the politics*, *diplomacy*.*’* [Asia7]

Developing good relationships with proximal countries was seen as more important than helping more distant countries.

*‘We must support the nearest or the closer neighbouring country first because people cross border together*. *That’s why we support [neighbouring countries]*, *because we have cross-borders with them*.*’* [Asia6]

One interviewee discussed the role of politics, and how sensitive geopolitical relationships might prevent smooth running of epidemic investigation.

*‘Joint outbreak investigation*, *this is also very sensitive*, *because one country cannot do the investigation in the other country*, *this is politically very sensitive*. *But some countries we agreed to do together[…]*. *Normally they will try offering investigation in the border area…’* [Asia1]

Governments need to be persuaded that they will benefit from joining a network with shared goals.

*‘When you are within an institution like Africa CDC and you know what you want*. *The issue will be negotiating in favourable terms so that you can achieve your objective*. *That’s where diplomacy comes in*, *and that’s where the issue of negotiating the outcomes of the collaboration*. *Defining common objectives*, *and that’s why it comes*, *it comes in*.*’* [Africa5]

Pragmatism is another important factor, recognising that countries are always going to be interested in how they benefit from membership.

*‘I think it’s more about understanding the country needs than hoping that a country will shift their needs to suit the agenda of the organisation*. *And I think that’s the only way that you can work with countries*. *Every country has their own political system*, *and sort of*, *I guess*, *then*, *you know*, *what the*, *what the authorities are interested in working on*. *And so you may*, *for example*, *in any given period*, *have at regional level*, *a wish or a hope that you can expand on a certain programme*, *but if there’s not an appetite for that in a country*, *then there’s nothing you can really do*. *I mean*, *of course*, *you can attempt to negotiate but I think it’s less successful than if you were understanding first the needs of the country and then trying to support them*.*’* [Europe1]

#### Ownership and sovereignty

The importance of member ownership was highlighted by two interviewees with experience in Africa, who emphasised that this encouraged sustainability as members realised how they benefitted.

*‘I love that all members own the network*. *I mean*, *let them treat it as their own*. *So that they*, *they really*, *I mean*, *is for their benefit and not for the network’s benefit […]*. *You really need to engage the decision-makers*, *make sure that institutions which are members actually know what the network is all about and how resources are being mobilised and how are they going to spend*, *rather than on individual basis because most of these networks were established on friendly basis*. *I know somebody*, *I would like to start something*, *are you interested*, *that’s the way things have started*, *but I want to make sure that they are sustainable*. *We need to make sure that the institutional members own the network*.*’* [Africa1]

Interviewees stressed that involving all members from initiation is essential to them feeling a sense of ownership, particularly for health ministries.

*‘You need to make sure that it is something that is owned by the member states*. *The ownership is something important*. *So*, *during the processes of establishing it you need to make sure that the member states are very closely involved*, *and when I say the member states I mean all key stakeholders*, *but mainly the ministries of health*, *they really need to be at the centre of the discussions*, *leading to the establishment of the centre*. *Ownership is something very important*.*’* [Africa5]

Sovereignty was discussed in many interviews. Interviewees recognised the importance of collaboration but stated that countries had to be responsible to their own populations before addressing issues in the region, as highlighted during the Covid-19 pandemic.

*‘We must do our best to protect the people in [our country] first and later on we expand our support to other country*.*’* [Asia6]

One suggested that a regional disease control body should not try and prevent countries behaving autonomously but instead should have a supporting role and provide guidance.

*‘I hope that the regional centre is not trying to do the job on behalf of the 10 countries*. *They should make themselves as the service to strengthen the centre in every country*. *So*, *it’s not a US CDC of that kind*, *but it’s a centre to support other countries*. *I don’t know whether and what direction they’re going*. *One direction is acting like the EU that comes out with a single standard*, *a single recommendation*.*’* [Asia5]

Even during epidemics, politicians may be reluctant to act and potentially undermine both in-country and regional emergency response efforts.

*‘Many countries have actually very good emergency response programs for emerging infectious disease[…]*. *Maybe they’re not as good as it should be*, *but it’s there*. *But to implement that policy is a political decision*. *Not a public health decision*. *It’s a political decision*, *has to be the politicians that say*, *yes*, *we’re going to take this seriously and respond to it*, *and politicians by nature*, *don’t like to do anything that is not definite*.*’* [Asia9]

#### Partnership development and collaboration

All interviewees noted collaboration and partnership-building as key to the success of any regional body or network. Referring back to the issue of trust, they described successful collaboration as involving face-to-face meetings, exchanging ideas, and regular visits to get to know collaborators and contexts.

*‘Trust is not coming by signing an agreement together*. *Trust only comes when you work and you see your friend*, *that talk*, *and the action goes in the same way[…]*. *I strongly encourage that current member countries in ASEAN need to have an exchange of staff*, *exchange of laboratories*, *people*, *from one country to work with other countries*. *By this you are forming a group of friends*, *and trust will come along*.*’* [Asia5]

Trust-building was described as better in person, although this was difficult during the Covid-19 pandemic.

*‘We have made like kind of the family [*…*] if they do*, *only in the online or something*, *email*, *they don’t know each other and that’s a little bit difficult for the working*. *So we just develop first*, *develop the trust*, *and making like a family feeling*. *So*, *working like a family and meeting two or three times so we’re just like a friend*, *so easy to work*, *and then easy to implement*. *Sometimes*, *even in the sensitive information*, *they can share with informally and they’re working together*.*’* [Asia1]

Interviewees discussed the importance of building relationships and infrastructure in normal times so collaboration can be leveraged immediately during a crisis.

*‘During peacetime you have to do the networking and tech transfer*. *Then*, *during the COVID-19 day you just put everything into action*.*’* [Asia7]

Interviewees suggested that capacity should be built in each country, with a division of labour so each country has its own infrastructure that can be leveraged during regional crises.

*‘Every member country should have their own disease control centres that are networking together to improve their own country capacities*. *So*, *if you have a very strong network of the member country*, *then moving the centre from one country to another country should not be a big problem […]*. *It should be that you try to promote training centre in every country*, *and you try to help them*, *how they’re going to use their own training centre for developing staff of their own country*.*’* [Asia5]

This also promotes a sense of participation and ownership, as all countries contribute.

*‘They can divide the diseases […]*, *one country is a leader in one thing*, *and he has to ask the other country for help if the other country is leader in something else*.*’* [Asia12]

Countries should have their own national focal person, responsible for administration in their country and contributing to wider regional priorities.

*‘We have in each country what we call chapters*, *national chapters*, *and the ones who do the coordination at country level*. *So*, *the headquarters which is in [country] coordinates the activities of the national chapters*.*’* [Africa1]

Having a good understanding of which stakeholders are already active in the geographical and thematic area is also important and helps ensure against duplication of effort.

*‘I think it’s very important to have a clear map of all actors involved in health emergency in Southeast Asia*. *Because when you have a good mapping of who is doing what as part of your business plan*, *you will definitely develop your strategy and your work plan*, *taking into account existing stakeholders and make sure that you are really adding value*, *not duplicating*. *So the mapping of stakeholders is very important*, *I guess*, *and*, *not only mapping*, *but also involving those stakeholders in the processes of establishing the new structure*.*’* [Africa5]

#### Political commitment and data sharing

The importance of having politicians involved from the beginning, with the associated political and administrative support–including belief in its aims, was highlighted by many interviewees.

*‘We can develop the best public health measures in the world*, *but unless the politicians*, *the policymakers are on board with it*, *and then truly believe in prevention*, *instead of response*, *it won’t work […]*. *We need to have real time communication between the health ministries in all of these countries and action that follows up*, *not just communication but there has to be action*. *That is a political problem*. *And so you have to bring in the politicians into these policy*, *the policy makers into these decisions*. *And the end of the day*, *it has to be a political organisation*, *which is difficult*.*’* [Asia9]

*‘The very first thing when we think of regional health organisation*, *it has to be political buy in from the countries*. *Because when we talk of surveillance and response*, *the data of the countries goes through that*. *So*, *if the countries are comfortable in doing that*, *with a clear objective of what it is going to help*, *and how this is going to benefit the people…’* [Asia14]

One interviewee suggested that governmental support might require pressure, and this could be done by involving the private sector, as governments would not want to be seen as not leading such an initiative.

*‘If you somehow devise a mechanism whereby*, *especially in health sector and veterinary sector*, *One Health sector*, *there’s an opportunity for involving the private institutions*, *and that can somehow put the social pressure on the government officials of each country to move forward*.*’* [Asia12]

Data sharing at national and regional levels is an essential component of any infectious disease control body and was discussed by many interviewees, with trust being perceived as key. One respondent talked about this geopolitically, equally applicable to any regional body.

*‘We talk a lot about sharing*, *but still I know […]*, *if an outbreak happens in India nobody tells you*. *Nobody informs Pakistan*. *Similarly*, *we have a problem*, *try to hide it*, *never tell India that this is what happened*. *So these are because of*, *I think*, *lack of confidence you can say*, *or[…]it may scare that if you’re too honest*, *you will be somehow*, *making trouble for me*, *that insecurity[…]That’s why people in the smaller side scale and the higher level*, *at the government level hesitate to share data and reporting*.*’* [Asia12]

Similarly, an interviewee discussed the effect that acknowledging a national outbreak may have on national economies, such as tourism and agriculture sectors.

*‘You have to share information*, *but the information is so sensitive*. *Yeah*. *Because if you inform that you have disease outbreak in your country*, *then the tourism is affected—the export of food may be affected*. *And that is why countries are reluctant to share information*. *We see this clearly on the avian influenza in the past*.*’* [Asia5]

### Organisation and management

Sub-themes were: (i) integration and cross-border challenges; (ii) financing and sustainability; and (iii) internal communication and capacity-building.

#### Integration and cross-border challenges

The major organisational challenge highlighted by many interviewees was how to integrate multiple countries with different cultures, languages, expectations and resources, financial backing, political support and laboratory and technical capacity, into a coherent body or network.

*‘I must say initially it was very difficult*, *because we’re bringing people from different countries with different cultures and backgrounds […]as we moved on*, *we started for instance having terms of reference*, *rules of engagement and so forth and then with time we got to know each other*, *our strengths and weaknesses*. *So it was a learning process’* [Africa2]

*‘Countries have different geographies*, *different politics*, *different requirements*, *different social circumstances*. *So*, *harmonizing all these is a challenge’* [Asia14]

Overcoming cross-border issues, and navigating work in borderland areas, given sometimes sensitive political relationships with neighbouring countries, was discussed by many interviewees.

*‘If their neighbours get affected*, *they are going to get affected*. *So putting politics aside*, *putting disputes on the side*, *I think health for everybody*, *for my people and for my neighbours*. *This is what’s important […]*. *We don’t talk about politics*, *we talk about humans here*, *and this is what’s important*.*’* [Asia8]

Many described the ASEAN region as particularly heterogeneous, with diverse capabilities that some suggested might pose issues. One suggested that if a regional cooperative body worked in ASEAN, it would work anywhere.

*‘For ASEAN*, *I’m thinking that because 10 member countries*, *that are quite varied*. *We have high-income country*, *middle-income country […]*, *we also have low-income country*. *ASEAN is a mini globe because its variety among 10 country—in terms of economy*, *in terms of geography*, *with mainland*, *and many islands in Indonesia*, *Philippines […] If anything can be successful within ASEAN*, *I think the world can be successful as well*. *Because it’s like as I said*, *it’s a mini world*.*’* [Asia6]

#### Financing and sustainability

Many interviewees discussed financing and sustainability in detail, with the lack of sustainable funding perceived as a serious impediment to any successful regional body. Some suggested that one country or respected sponsor should lead on financing, both to allow them to use their expertise and to encourage other investors.

*‘You need to have some country like Singapore*, *or another major body*, *say “Yes*, *this is important*,*” that we’re going to take this*, *we’re going to help fund it*. *And then that will encourage other people*, *other funders*, *to come in and join*, *but you’re going to have to put together a good sum of money in order to get this off the ground…The issue there is again funding*. *And so you’re going to have to solve that problem*. *The best way to do that is to get a highly respected sponsor to help you raise the funds and convince people to buy into this’* [Asia9]

Another underlined this by advocating for assessed contributions and higher-income countries contributing more than lower-income ones.

*‘Funding and sustainability is a major question […] unless there is a buy-in from the countries […] assessed contribution of the countries from the GDP*, *plus some voluntary contribution by those countries which have capacities to pay for others*.*’* [Asia14]

As funding is often given by donors, rather than national governments, organisations must work within donor interests. When donor funding ends, projects also end, even if they focus on something essential such as disease surveillance.

*‘Often there’s no funds from the government for the disease surveillance*, *so it only happens when an outside donor comes in*, *right*? *And then once that donor stopped*, *it’s gone*.*’* [Asia2]

One interviewee suggested that organisations should ‘follow the money’, in that donors fund what interests them and resource-constrained organisations have no option but to focus accordingly.

*‘Controlling the funds is a huge factor*. *You know*, *it’s the golden rule*. *He who has the gold makes the rules*.*’* [Asia4]

Lack of secure funding makes planning difficult. Many interviewees discussed how unsustainable funding streams resulted in piecemeal projects that ended before outcomes were achieved and prevented strategic planning. Particularly for a regional body tasked with epidemic response and preparedness, long-term core funding is necessary.

*‘A steady stream of funding that would allow you to plan for your work and*, *you know*, *and not go from grant to grant and you know*, *challenge really in non-profits or organisations like this that receive external funding is that the funders often drive the subject matter that you are working on*. *So*, *one day you’ll work on malaria and the next day you work on health-worker training for something else*, *depending on what the funders interested in and so the danger to these things is that your long-term strategy may be difficult to implement because streams of funding come in for specific things that sort of make you change that*.*’* [Americas1]

Lack of sustainable funding means little job security, so staff may work on short-term contracts then leave, making any investment in their training and capacity building a financial loss.

*‘And then after those three or four years there*, *they leave again*, *you know*. *To just leave science or whatever […]it’s very difficult*. *You build the capacity*, *and then you have to rebuild it*. *And then you got to rebuild it*. *And then you’ve got to rebuild it*…*’* [Asia2]

One described a pragmatic mixed strategy of relying on well-resourced funders for large amounts and obtaining smaller items from local sources.

*‘What we did was that we decided to get difficult things from the funding agency*, *and the easy things from our local funding and stabilise things in a way that when you have the basic*, *equipment and buildings like already constructed*, *somehow by partial funding*, *renovation of building*, *purchased a vehicle and then the little funds*, *you can very easily get from the local governments*.*’* [Asia12]

Strengthening existing systems and projects can demonstrate what works, which is key for ensuring government awareness and funding support.

*‘For sustainability*, *what we’ve done was to strengthen the existing surveillance systems and not to embark on new ones*, *because that may not be sustainable beyond [our network]*. *And that has worked very well*. *And making certain small and demonstrated achievement has made the government know some of the issues’* [Africa2]

#### Internal communication and capacity-building

The Covid-19 pandemic affected how organisations communicated, with online discussions replacing face-to-face interactions. This had the positive effect of involving stakeholders and experts from other regions or who did not have funds for face-to-face visits.

*‘The webinars are organized by the network*. *We […] support them to organize a webinar in different topics*, *mainly about COVID in the last year*. *And so there is a lead network*, *and there are*, *you know*, *invited speakers from other networks or from experts from other parts of the world*, *and this was very successful with over 200 from the network’* [Americas2]

Meeting as a large group is not always practicable, or necessary, with one interviewee advocating the effectiveness of smaller, more frequent meetings on similar themes or projects.

*‘Before the Covid pandemic we were meeting once or twice a year*, *physically*, *and then in between we will communicate via emails*, *and then came Zoom*, *I mean*, *which makes our life easier*, *so we have quite frequent Zoom meetings*, *and in many cases*, *it is COP [community-of-practice] meetings*, *not the entire [group]*.*’* [Africa2]

Other communication methods included newsletters and social media.

*‘The other activity that we maintain is issue a newsletter quarterly*, *where we publish*, *each network again contributes a note or piece*, *not an article*, *really*, *a note*. *And we also have a working group on communications*, *a communications plan that includes their website*, *their social media channels…’* [Americas2]

Lack of capacity can make a bad situation worse, and affect neighbouring countries, underlining the importance of countries sharing information and surveillance training to protect the region.

*‘So the idea is that countries should be able to identify early and intervene early*, *at the source of the outbreak limiting its spread*. *But we know that countries are in different level of development of their public health services and that’s not always the case*. *So*, *if we miss this opportunity*, *an outbreak becomes extended at a country or regional*, *subnational*, *or national level*.*’* [Americas2]

*‘I think our biggest challenge today to develop the […] Asian CDC*, *is to build laboratory and epidemiologic capacity that allows us to develop proactive surveillance*, *that allows us to detect and identify and contain viruses before they start to spread’* [Asia9]

Variations in capacity (e.g. financial, staffing, technology) between countries was highlighted as a potential barrier to developing any regional body, but also as an opportunity to share best practices and increase capacity. Capacity building was identified as a way for countries to develop strong relationships, as all could share what they know and do best.

*‘We have been promoting for joint outbreak investigation and capacity building*, *and also the tabletop exercise*, *since 2004[…]*. *And then the risk assessment workshop*. *So*, *especially we have been doing for the capacity building for the cross-border area and annual regional forum*. *So we invite all the cross-border people to attend*, *then explain about their experience and their challenges*, *sharing with each other*, *meeting each other’* [Asia1]

One element of capacity building is increasing the ability of subnational staff to manage large budgets.

*‘So if you have an NGO anywhere in Asia or Africa they might have a couple of million dollars*. *It’s the old story*, *you put too much water in the balloon it’s going to pop*, *they just can’t handle 20 or 30 million dollars a year and it’s hard to build up the capacity for that from a financial management point of view’* [Asia4]

Interviewees stressed that while science/technical capacity was crucial, so were infrastructural and managerial capacity.

*‘It takes years to create infrastructure networks*. *And we’ve focused on the science and all that*. *But what I’ve learned in my career is equally important*, *really is*, *your management capacity*, *the ability to manage awards*, *contracts and grants in the financial management of things’* [Asia4]

### External partnerships and engagement

Sub-themes were: (i) multilateral partners; and (ii) public engagement and external communications.

#### Multilateral partners

Any regional disease control body will have to collaborate with multilateral organisations, such as WHO or the World Bank, to ensure engagement and secure financing. There will always be external relationships to negotiate, and members must be pragmatic about this. Many interviewees discussed the potential role of WHO in facilitating or helping convene any regional health body.

*‘I don’t think you would succeed without WHO support*. *But*, *you know*, *with an organisation like ASEAN behind you*, *I think*, *then that helps cut across*. *It doesn’t mean that WHO shouldn’t be involved*, *but also it can be a coordinating body that could really help Asian CDC’* [Asia9]

*‘Another option could be like secondment or maybe like ASEAN CDC staff may be seconded to WHO SEARO or WPRO*, *for let’s say one year*, *two year*, *maybe vice versa*, *WHO staff can be seconded to the ASEAN CDC[*…*]they can borrow some systems or procedures from WHO so this kind of human exchange might be useful’* [Asia10]

Another interviewee suggested that multilateral organisations could potentially be responsible for enacting and enforcing policy to prevent infectious disease spreading, as this should not be left to national governments.

*‘I mean*, *if we can legislate*, *you know*, *and have an international law*, *that would be the ultimate kind of position to fight any future pandemics right if we can achieve that*. *For example*, *the law says that*, *you know*, *once you have a class stuff on known pathogens*, *you know*, *cause human death in a severe disease*, *then the regional body or the international body have the right to arrive*, *anytime at their kind of choice and to work*, *collaborate with the national team*, *to have a rapid response team basically right[…]you have to have an international or regional network and expertise team can just go in and then help[…]right now it’s at the mercy of local government to say you’re welcome or you’re not welcome*.*’* [Asia7]

#### Public engagement and communications

The role of communities is often neglected, despite being the intended audience for most policy. Interviewees highlighted that it is not enough to disseminate scientific data, issues must be explained in a relevant way to citizens.

*‘The users*, *most of the time are left behind*. *I mean you expect them to adopt*, *especially for us who are working as researchers*, *you expect users to adopt easily because there is scientific evidence*. *I don’t believe in that*. *I think we need to bring them on board*, *you know*, *conceive the ideas*. *Okay*, *we’ll do the implementation but then they’ll be able to follow up*. *How were the products or findings generated*, *and they know they know how to bring them down to the community*. *I mean to the end users*. *Most of us don’t have a lot of skills in advocacy’* [Africa1]

The importance of engaging communities at all stages of building a regional disease control body was underlined by two interviewees.

*‘I think one of the weaknesses*, *we have is that most often these networks are researcher networks*, *and then we*, *the decision-makers*, *policymakers*, *rarely get involved and this makes other institutions having lots of difficulties in*, *I mean*, *providing inputs and participating fully*. *So I think it’s important that we really bring various key stakeholders at different levels on board*, *so that whatever you generate is being adopted by the end users’* [Africa1]

Another discussed the importance of meaningful risk communication.

*‘Risk communication involves working with different level community partners for community engagement*. *We develop SOPs [standard operating procedures]*, *develop guidelines*, *develop strategies that states adapt to respond*. *So we are more like that coordination level*, *churning out knowledge*, *engaging the different stakeholders and making sure that the states and the relevant development partners have the needed information to respond to infectious disease outbreaks’* [Africa3]

One interviewee gave an example of the importance of involving communities in regional projects, as, without this ‘on the ground’ awareness, projects will likely fail.

*‘We were looking at the impact of agriculture on malaria*, *[…]away we went to the community*. *Before we started*, *we had meetings with the local community*, *the local leaders*, *and you know what happened*? *They said ‘Oh*, *you guys who told you malaria is a problem in our community*? *Our problem here is food and schistosomiasis*. *If you can work on that*, *go ahead*. *If you can’t*, *please pack and go*.*’ So we had to revise our protocol to include those two issues*, *and the project ran for over 6 years very*, *very successful*. *Everybody was involved at all levels’* [Africa1].

The Covid-19 pandemic also highlighted the role of the public and communities in infectious disease responses.

*‘The investment should move towards community*, *making sure that the*, *the surveillance system*, *the health system at the community level is well resourced*, *we have been relying so much on our volunteer workers*, *community health-workers but now we’re realising that they play a very vital role*, *especially for COVID*, *because they do the contact tracing*, *they do the advice*, *they give advice*, *they do the case detection[…]and they refer patients*, *so these are very crucial’* [Americas3]

*‘The example of home isolation and community isolation is a good example for community engagement and participation of the people in Thailand*. *Everyone counts*, *because everyone complies with the campaign of wearing masks*, *social distancing and temperature measure often*, *and also the hand hygiene*. *Everyone is important*, *and then we need cooperation*, *by everyone in the country’* [Asia6]

## Discussion

To our knowledge, this was a first attempt to synthesise perspectives on operationalising infectious disease control cooperation in different regions. Governmental and public awareness of the potential spread of infectious disease, exemplified by the Covid-19 pandemic, may increase opportunities for regional infectious disease control cooperation. However, it remains challenging to implement [20].

Our findings support those of our earlier scoping review, suggesting key drivers for successful implementation of a regional body, such as the newly established ACPHEED, include ensuring inclusion of major stakeholders throughout the process and promoting capacity building in member countries helped to ensure sustainability [20]. Our interviews pinpointed three main areas that global experts believed are essential for such a body to function effectively. These were (i) strong governance; (ii) effective management—particularly of sustainable funding; and (iii) working with global, regional, and national stakeholders, including the public. Integrating all member countries within the organisation, promoting a feeling of ownership, increasing staff capacity through training and exchanges, and clear communication were also highlighted as necessary for any regional organisation to succeed. Some interviewees discussed personal experiences, reflecting on what did or did not work in their organisations’ operationalisation. Others underlined the importance of understanding the current situation and then planning strategically what the putative organisation should aim to achieve.

One issue that regularly arose in the interviews was the importance of working with neighbouring rather than distant countries, partly to control the risk of importing or spreading disease but also in terms of enlightened self-interest. This is a key factor for an organisation for which, as some interviewees stated, geographical proximity is likely to be the only or primary linkage. There was a perception that countries were more likely to collaborate with and assist those who could more easily offer assistance in turn, or those countries with whom a government has close diplomatic connections. This perception means that governments must be encouraged to work with disparate countries with whom they may not have culture, language, or political economy factors in common. How to foster this type of work was considered by many of our interviewees, with agreement on the importance of building relationships by involving all parties from inception, to promote ownership and encourage discussion of potentially sensitive issues.

Regional geopolitics may complicate relationship-building and must necessarily be considered, particularly in regions with a range of resource-poor and richer countries [5]. Interviewees commented that local political sensitivities can complicate aspects such as data sharing, which country manages projects that involve working across borders, and political will, as governments may not want to admit that their country has an outbreak [6–8]. Acknowledging the existence of an infectious disease outbreak may affect business interests, as was recently demonstrated in South Africa [9]. Thus, further complications to building effective regional collaborative organisations include putative effects on tourism and trade, as seen during the ban on travel to South Africa when Omicron was first detected there [10] and also on the international food trade, exemplified by a report from the Food and Agriculture Organization of the United Nations [23]. This report suggests that countries should work together to prevent disruption to global food production and supply chains. This can only be done by leveraging pre-existing relationships that have built trust.

Building capacity, and supporting knowledge transfer both within and between countries, promote goodwill and staff retention, which in turn promotes organisational effectiveness. Regular monitoring and evaluation of activities is key to measuring success, and successes can be used to encourage staff and demonstrate to funders the importance of what the organisation is trying to do. This starts a virtuous cycle, whereby success inspires confidence in the organisation and funders are more likely to disburse further funding.

Several limitations should be considered. Most interviewees were male and most judged their experience to be related to Asia, with only one stating her expertise involved Europe. Thus, perspectives reflect those of regional infectious disease communities, which are often male dominated, rather than necessarily drawing from more marginalised experiences. Future research could help address this, although our findings indicated that interviewees shared many of the same beliefs and concerns, despite their perceived gender or geographical expertise. Some invitees did not participate, which is perhaps unsurprising given the COVID-19 pandemic context. While findings should be interpreted accordingly, we did not note any obvious ‘responder biases’ and were still able to achieve sufficient data saturation.

## Conclusion

The international infectious disease community has learned valuable lessons from the Covid-19 pandemic, not least the necessity of pooling human, financial and technological resources, constructing positive working relationships with other countries in the region, and sharing data. Without this kind of regional cooperation, infectious diseases will continue to threaten our future, and the next pandemic may have even more far-reaching effects than Covid-19. The insights from our interviews support our earlier findings: operationalisation of a regional infectious disease body is complex but focus on areas like the three themes that our interviewees highlighted–organisational factors; governance and diplomacy; and stakeholders–will help promote successful initiation and implementation.

## Supporting information

S1 Checklist (PDF)
